# The Present and Future of Prostate Cancer Urine Biomarkers

**DOI:** 10.3390/ijms140612620

**Published:** 2013-06-17

**Authors:** Marina Rigau, Mireia Olivan, Marta Garcia, Tamara Sequeiros, Melania Montes, Eva Colás, Marta Llauradó, Jacques Planas, Inés de Torres, Juan Morote, Colin Cooper, Jaume Reventós, Jeremy Clark, Andreas Doll

**Affiliations:** 1 Research Unit in Biomedicine and Translational Oncology, Vall d’Hebron Research Institute and Hospital and Autonomous University of Barcelona, 08035 Barcelona, Spain; E-Mails: marina.rigau@vhir.org (M.R.); mireia.olivan@vhir.org (M.O.); marta.garcia.lopez@vhir.org (M.G.); tamara.sequeiros@vhir.org (T.S.); melania.montes@vhir.org (M.M.); eva.colas@vhir.org (E.C.); marta.llaurado@vhir.org (M.L.); jaume.reventos@vhir.org (J.R.); 2 Department of Urology, Vall d’Hebron University Hospital and Autonomous University of Barcelona, 08035 Barcelona, Spain; E-Mails: jplanas@vhebron.net (J.P.); jmorote@vhebron.net (J.M.); 3 Department of Pathology, Vall d’Hebron University Hospital Autonomous University of Barcelona, 08035 Barcelona, Spain; E-Mail: itorres@vhebron.net; 4 Cancer Genetics, University of East Anglia, Norwich Norfolk, NR4 7TJ, UK; E-Mails: colin.cooper@uea.ac.uk (C.C.); jeremy.clark@uea.ac.uk (J.C.); 5 Department of Basic Sciences, International University of Catalonia, 08017 Barcelona, Spain

**Keywords:** prostate cancer, biomarker, urine, non-invasive

## Abstract

In order to successfully cure patients with prostate cancer (PCa), it is important to detect the disease at an early stage. The existing clinical biomarkers for PCa are not ideal, since they cannot specifically differentiate between those patients who should be treated immediately and those who should avoid over-treatment. Current screening techniques lack specificity, and a decisive diagnosis of PCa is based on prostate biopsy. Although PCa screening is widely utilized nowadays, two thirds of the biopsies performed are still unnecessary. Thus the discovery of non-invasive PCa biomarkers remains urgent. In recent years, the utilization of urine has emerged as an attractive option for the non-invasive detection of PCa. Moreover, a great improvement in high-throughput “omic” techniques has presented considerable opportunities for the identification of new biomarkers. Herein, we will review the most significant urine biomarkers described in recent years, as well as some future prospects in that field.

## 1. Introduction

Cancer is one of the most critical health problems in our society, both in terms of morbidity and social impact. Prostate cancer (PCa) is the most commonly diagnosed cancer among European and American men (29% of all cases) [1,2]. Although PCa is a slow growing tumor that affects older men, it is still a lethal disease and is currently the second most common cause of cancer death among men [2]. The long latency period of this type of cancer and its potential curability make this disease a perfect candidate for screening [3].

Current screening techniques are based on a measurement of serum prostate specific antigen (*PSA*) levels and a digital rectal examination (DRE). A decisive diagnosis of PCa is based on transrectal ultrasound-guided prostate biopsies (PBs). The use of serum *PSA* as a cancer-specific detection test has some well-recognized limitations, such as a low positive predictive value (PPV).

When *PSA* is 4.0–10.0 ng/mL, the PPV is 18% to 25% (mean, 21%), and when *PSA* is >10 ng/mL, the PPV is 58% to 64% (mean, 61%), when combined with a DRE as a screening tool this still results in approximately 66% negative PBs [4–6]. These patients are often subjected to repeat *PSA* measurements and PBs (the “over-diagnosis” problem). “Over-treatment,” through the detection of non-life-threatening tumors [7], especially in the so-called gray zone (serum *PSA* between 4–10 ng/mL), represents yet another dilemma, as it is difficult to discriminate between patients with PCa and those with benign prostatic hyperplasia (BPH) or between those patients suffering from prostatitis and the results of urethral manipulation, which can also increase *PSA* levels [8]. Conversely, the prevalence of PCa in patients with *PSA* levels below the threshold of 4 ng/mL is around 15% resulting in undiagnosed cases of the disease [9,10]. As a consequence of the current screening parameters, approximately two thirds of the 1 million biopsies made annually both in the United States and in Europe are unnecessary [1,2]. There is therefore an urgent need for new and more effective biomarkers for PCa that can help to better identify which patients should undergo further diagnostic tests and also help to detect which patients will develop an aggressive tumor and, therefore, will need immediate treatment.

## 2. Urine: A Source of Prostate Cancer Biomarkers

The discovery of biomarkers is based on the following research principle: the comparison of physiological states, phenotypes or changes across control and case (disease) patient groups [11]. A key approach to biomarker discovery is to compare case versus control samples in order to detect statistical differences that can lead to the identification and prioritization of potential biomarkers. Theoretically, this could be a single biomarker molecule, however, it is more likely to be a panel of up- and down- regulated molecules and/or proteins with altered post-translational modifications (PTMs) that differ in normal and disease states [12,13]. Here we have focused our biomarker classification system on the basis of their potential applications for screening, diagnosis, prognosis or prediction (see Box I).

Box I **Types of Biomarkers Based on their Applications**

Screening/detection biomarkers, like serum *PSA*, are used to predict the potential occurrence of disease in asymptomatic men or those with non-disease-specific symptoms.

Diagnostic biomarkers are used to make predictions for patients suspected of having a disease. An ideal diagnostic biomarker should enable an unbiased conclusion, particularly in patients without specific symptoms. It should fulfill several criteria: (i) high specificity for a given disease (low rate of false positives); (ii) high sensitivity (low rate of false negatives); (iii) ease of use (rapid procedure); (iv) standardization (consistent reproducibility); (v) clearly readable result for clinicians [13]; (vi) cost-effectiveness; and (vii) ability to be quantified in an accessible biological fluid or sample.

Prognostic biomarkers are used to predict the overall outcome of a patient, regardless of therapy.

Predictive biomarkers are used to identify subpopulations of patients who are most likely to respond to a given therapy. A predictive biomarker can be a target for therapy.

In recent years interest in searching for new biomarkers obtainable by non-invasive means has increased significantly. For centuries, physicians have attempted to use urine for the non-invasive assessment of disease. Urine is produced by the kidneys and allows the human body to eliminate waste products from the blood. Urine may contain information not only from kidney and urinary tracts, but also from distant organs via plasma obtained through glomerular filtration. The analysis of urine could, therefore, allow the identification of biomarkers for both urogenital and systemic diseases.

**Figure 1:**
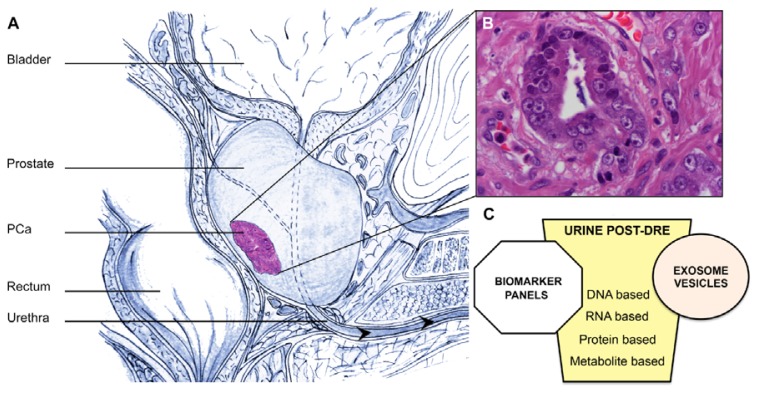
(**A**) Anatomical location of the prostate; (**B**) Prostate cancer cells; (**C**) Biomarkers found in urine. Based on their descriptions, biomarkers can be divided into the following groups: DNA-based, RNA-based, and protein-based. Of late, urinary exosomes, which are secreted vesicles that contain proteins and functional RNA and miRNA molecules, have emerged as a novel approach to acquiring new PCa biomarkers.

The main function of the prostate gland is the secretion of prostatic fluid, which on ejaculation is combined with seminal vesicle derived fluid to promote sperm activation and function [14]. The gentle massage of each side of the prostate gland during DRE stimulates the release and movement of prostatic fluids and detached epithelial cells into the urethra [14] (Figure 1). These fluids can contain both cells and secretions originating in PCa [15]. PCa cells were first described in voided urine by Papanicolaouin 1958 [16], however they appear to be fragile and low in number [17] underlying the need for careful collection, manipulation and storage of urine prior to analysis. Urine collection can be accomplished without a disruption of standard clinical practice and can be sampled multiple times throughout the course of prostatic disease. Nevertheless, using urine for the discovery of biomarkers presents some important technical challenges.

The search for effective biomarkers has principally included transcriptional profiling, DNA methylation, metabolomics, fluxonomics, and more recently, proteomics [18]. Emerging biomarkers have the potential to be developed into new and clinically reliable indicators, which will have a high specificity for the diagnosis and prognosis of PCa. Ideally biomarker acquisition will be less invasive than current clinical means, and will be useful for screening men for PCa, and be able to guide patient management to provide maximum benefits while minimizing treatment-related side effects and risks [19]. This review focuses on published data referring promising DNA, RNA, miRNA, protein and metabolite based urine biomarkers (Table 1) and highlights exosomes as a new source of PCa urinary biomarkers.

### 2.1. DNA-Based Urinary Biomarkers

DNA-based biomarkers include single nucleotide polymorphisms (SNPs), chromosomal aberrations, changes in DNA copy number, microsatellite instability, and altered promoter-region methylation [109]. The epigenetic silencing of the glutathione-*S*-transferase P1 (*GSTP1*) gene is the most common (>90%) genetic alteration so far reported in PCa [110–112]. Methylation-specific polymerase chain reaction (MSP) methods allowed the successful detection of *GSTP1* methylation in urine, and ejaculates from PCa patients. A possible drawback is the high frequency of *GSTP1* methylation in patients with high-grade prostatic intraepithelial neoplasia (HG PIN) and in patients with negative or suspicious PB. Further follow-up is needed to determine whether such cases are false positives or part of the significant number of under-diagnosed cancer cases in PB. Recently, Costa *et al.* observed significantly different methylation levels of the genes protocadherine 17 (*PCDH17*) and transcription factor 21 (*TCF21*) in PCa tissue compared to cancer free individuals, providing 83% sensitivity and 100% specificity for cancer detection. However while absolute specificity was retained in urine samples, sensitivity was only 26% [113]. In comparison, Daniunaite *et al.*, (2011) report the high sensitivity of DNA methylation biomarkers in urine, especially that of *RASSF1* (Ras association (*RalGDS/AF-6*) domain family member 1) and *RARB* (retinoic acid receptor beta) for the early and non-invasive detection of PCa. Thus, results this far suggest that methylated genes can serve as useful markers for PCa [97].

### 2.2. RNA-Based Urine Biomarkers

RNA-based biomarkers include coding and non-coding transcripts and regulatory RNAs, such as microRNAs (miRNAs) [109]. Improvements in RNA microarray platforms, quantitative PCR (qPCR), and the development of new high-throughput technologies, such as next-generation sequencing (NGS), allow us to better understand the expression profiles of single cells, populations of cells and specific tissues, while also allowing comparisons between different pathological conditions. In recent years, a wide range of promising PCa biomarkers that are not only prostate-specific, but also differentially expressed in prostate tumors, have been identified.

After *PSA*, Prostate Cancer Antigen 3 (*PCA3*), is the only biomarker approved by the Food and Drug Administration (FDA), and is utilised in a commercially available test under the name PROGENSA® *PCA3* (Gen-Probe, San Diego, CA, USA) [84]. *PCA3* was first identified in 1999 [85]. The *PCA3* gene encodes a non-coding RNA (ncRNA) (see Box II) that is over-expressed in 95% of all primary PCa specimens. Some of its potential applications include testing as an alternative to a first PB and, aiding the decision whether to repeat a PB in men with high serum *PSA* levels and previously negative biopsies [86,87]. The measurement of *PCA3* mRNA *vs. PSA* mRNA in urine was first proposed by Hessels *et al.* [88]. Later on, this study was verified in a large, European multicenter study, which concluded that *PCA3* possessed potential as an aid in PCa diagnosis [89]. The assay consists of a transcription-mediated amplification, which demonstrates 69% sensitivity, 79% specificity, and an area under the curve (AUC) value of 0.75 [90]. Currently, a PCA3 score (*PCA3*-to-*PSA* ratio) cut-off of 35 has been adopted, which combines the greatest cancer sensitivity and specificity (54% and 74%, respectively) [91]. However, more recent studies have shown that a lower cut-off score of 25 might be preferable [92].

Box II **Non-coding RNA**

A “central dogma” of molecular biology was that genetic information flowed in one direction with proteins as the end product. However, growing evidence has emerged to describe the role of RNAs that are not translated into proteins. These ncRNAs comprise microRNAs, anti-sense transcripts and other transcriptional units containing a high density of stop codons and lacking any extensive “Open Reading Frame” (ORF) [139]. Several types of ncRNAs have been implicated in gene regulation via modification of the chromatin structure, alterations to DNA methylation, RNA silencing, RNA editing, transcriptional gene silencing, post-transcriptional gene silencing, and enhancement of gene expression [140–142]. It is becoming clear that these RNAs perform critical functions during development and cell differentiation [139]. The roles that small-ncRNAs, such as miRNAs and small interfering RNAs (siRNAs), play in gene silencing have been well-studied, and they have been reported to be aberrantly expressed in many cancers [140]. ncRNAs are thus emerging as a new class of functional transcripts in eukaryotes.

Prostate Specific Membrane Antigen (*PSMA*) was first proposed as a serum prognostic marker for PCa in 1999; however, its use is controversial [114]. A Dual-Monoclonal Sandwich Assay for *PSMA* was developed to be used on tissues, seminal fluid and urine [115]. Levels of *PSMA* in serum have been suggested to be useful for distinguishing between BPH and PCa [116], and subsequently the same results were found for urinary PSMA [117]. *PSMA* is present in exosomes in urine samples from PCa patients after therapy [118]. Our group has evaluated the utility of *PSMA* mRNA transcripts in conjunction with *PCA3* and Prostate Specific G-coupled Receptor (*PSGR*) in the *PSA* diagnostic “gray zone” of 4–10 ng/mL when no prior biopsy information was available. We demonstrated that the prediction of PCa improved significantly for *PSMA* (0.74), while *PSGR* (0.66) and *PCA3* (0.61) showed a similar performance [119]. However, the use of *PSMA* has not yet been adopted in clinical practice.

Another promising RNA-based urinary biomarker is encoded by a fusion gene formed as a result of a translocation between the androgen-regulated transmembrane protease, serine 2 (*TMPRSS2*) gene transcriptional promoter and the ETS related oncogene *(ERG)*, resulting in an androgen-regulated *TMPRSS2–ERG* fusion gene that is highly specific for PCa and can be found in approximately half of all white PCa patients [120]. Hessels *et al.*, analyzed *TMPRSS2-ERG* fusion transcripts in urinary sediments and demonstrated a sensitivity of 37% and a specificity of 93% for the prediction of PCa [104]. Moreover, *TMPRSS2-ERG* was correlated with pathological stage [121], Gleason score [121,122] and with PCa death [122]. Additional marker analysis in a multiplex detection system could further improve sensitivity and specificity.

### 2.3. miRNA-Based Urine Biomarkers

The discovery of miRNAs has opened up a new field in cancer research with potential novel applications in diagnostics and therapy [123]. MicroRNAs are short, ncRNAs with an average length of 22 nucleotides [124] (see Box II). After transcription they fold into hairpin structures before being processed into mature miRNAs that bind to complementary sequences in mRNAs to alter protein expression. Currently, 1600 precursor and 2042 mature human miRNAs are registered in miRBase Release 19 (August 2012), and each of these may target up to 1000 gene sequences [125]. This provides a complex layer of control in for example, signaling pathways involved in the regulation of cellular functions, ranging from the maintenance of “stemness” to differentiation and tissue development, and from the cell cycle to apoptosis and metabolism [126–128]. Thus, aberrant expression of miRNAs can impact deeply on multiple features of cell biology resulting in complex downstream pathological events, such as cancer [129]. Specific miRNAs have been shown to be abnormally expressed in tumor tissues, playing important roles in cancer onset and disease progression through the targeting of cancer-relevant genes [130].

miRNA profiles of different tissues have been reported to be more predictive than mRNA characterization to such an extent that poorly differentiated tumors of uncertain origin could be classified on the basis of miRNAs expression [131]. MiRNAs are very stable and are detectable in biopsies, serum, and other fluids, such as urine [132]. Between 200 and 500 miRNAs were detected by qPCR in different human body fluids, such as plasma, urine and breast milk [133]. Mitchell *et al.*, found that the serum levels of the miRNA “miR-141” distinguished patients with advanced PCa from healthy controls [134]. Other recent studies have demonstrated that circulating miR-141 levels were correlated to aggressive PCa [135], and that miR-96 and miR-183 expression in urine were well correlated to urothelial carcinoma (UC) stage and grade, serving as promising diagnostic tumor markers capable of distinguishing between UC patients and non-UC patients [136]. However, only one study has been published linking miRNAs from urine with PCa. In that study, the analysis of five selected miRNAs in urine samples found that miR-107 and miR-574-3p were present at a significantly higher concentration in the urine of PCa patients compared to controls [137].

In PCa most of the circulating miRNA studies which have found associations between miRNA populations and aggressive and metastatic disease have been conducted using serum or plasma and need to be validated in larger patient and control samples [130]. Specific miRNA patterns in the urine may also reflect early or advanced PCa disease, but while urine miRNAs have been investigated in bladder and kidney cancer, no comprehensive studies for miRNA in PCa urine have been reported so far. Therefore, despite the obvious potential for circulating and urine miRNAs in diagnostic, prognostic, and predictive applications, clinical implementation of a non-invasive miRNA test for PCa is still a distant goal [138].

### 2.4. Protein-Based Urine Biomarkers

Protein-based biomarkers include cell-surface receptors, tumor antigens (such as *PSA*), phosphorylation states, carbohydrate determinants and peptides released by tumors into serum, urine, sputum, nipple aspirates, or other body fluids [109]. Proteins secreted by cancer cells can be essential in the processes of differentiation, invasion and metastasis [143,144]. Secreted proteins or their fragments present in body fluids, such as blood or urine, can be measured via non-invasive or minimally invasive assays. To date, only a few studies have analyzed cancer secretomes. However, the results with regards to the discovery of biomarkers are rather exciting [145].

Recently the detection of under-expressed *PSA* protein levels in urine has been reported [146–149]. Bolduc *et al.* compared a small cohort of urine samples collected (without previous DRE) from “normal”, BPH and PCa men, and the data suggested that the ratio of serum *PSA* to urine *PSA* could possess diagnostic value [146]. The same idea was also suggested in another independent study where *PSA* levels were also determined in urine. In that study, no differences between urinary *PSA* pre- and post-PM were found [150]. Later, Drake *et al.* [14] performed a study in which they focused on the characterization of *PSA* and Prostatic Acid Phosphatase (*PAP*) using an Enzyme-Linked ImmunoSorbent Assay (ELISA) assay on post-DRE urine samples. They found a clear trend towards lower levels of expression for both proteins in their cancer samples.

Another protein-based candidate is Annexin A3 (*ANXA3*), which is a calcium-binding protein with an associated decreased production in PCa cells. The analysis of *ANXA3* using Western blots (WB) of urine samples showed significantly lower values in PCa patients as compared with BPH patients. When this marker was combined with serum *PSA* there was improved sensitivity and high specificity compared to total *PSA*, with an AUC of 0.81 [151]. Katafigiotis *et al.*, looked at urine samples from 127 PCa patients obtained after DRE, measuring zinc α 2-glycoprotein (*ZAG*) by WB. Receiver operating characteristic (ROC) curve analysis showed a significant predictive ability for PCa with AUCs of 0.68 [32].

Recent advances in liquid chromatography (LC) and two dimensional gel electrophoresis (2D-GE), in combination with mass spectrometry (MS) have significantly facilitated the challenging detection of proteins in body fluids [152]. High-throughput proteomic analysis of biological fluids such as urine, has recently become a popular approach for the identification of novel biomarkers, due to the reduced complexity compared to serum [153]. However, only a limited number of studies have focused on PCa.

One of the first proteomic urine profiling experiments for the detection of PCa was performed by Rehman *et al.*, using a gel-based strategy comparing PCa and BPH samples [154]. They identified *S100A9* (calgranulin B, *MRP-14*) as a possible biomarker. However, this data was not verified in an independent study. More recently, several studies have focused on the characterization of urine samples in a high-throughput manner. Teodorescu *et al.*, performed a pilot study for PCa using Capillary Electrophoresis (CE) coupled with MS and to define a potential urinary polypeptide pattern with 92% sensitivity and 96% specificity [155]. Later, the same group described a refinement of the PCa specific biomarker pattern using 51 PCa and 35 BPH urine samples [156]. The model, containing 12 potential biomarkers, resulted in the correct classification of 89% of the PCa cases and 51% of the BPH cases in a second blind cohort of 213 samples. The inclusion of age and free *PSA* parameters increased the sensitivity and specificity to 91% and 69%, respectively. M’Koma and collaborators performed a large-scale proteomic analysis of BPH, HGPIN and PCa urine samples [157]. Using Matrix Assisted Laser Desorption Ionization-Time of Flight (MALDI-TOF) analysis, the group reported 71.2% specificity and 67.4% sensitivity for discriminating between PCa and BPH, while they also reported a specificity of 73.6% and a sensitivity of 69.2% for discriminating between BPH and HGPIN. Finally, Okamoto *et al.* used Surface Enhanced Laser Desorption Ionization Time of Flight (SELDI-TOF) analysis coupled to MS to analyze post-DRE urine samples. They obtained a heat map with 72 peaks, which could distinguish PCa from benign lesions with a sensitivity of 91.7% and a specificity of 83.3% [158]. However, although there have been an increasing number of publications in the proteomic urine PCa field, most of this data has not been verified in independent studies.

### 2.5. Metabolite-Based Urine Biomarkers

Metabolomics is a recently incorporated–omic approach that identifies metabolites using techniques similar to proteomics. Urinary metabolomic profiles have recently drawn a lot of attention owing to a debate regarding their possible role as potential clinical markers for PCa [159]. Using 262 clinical samples, including 110 urine samples, Seekumar *et al.* performed a major study in the field of PCa metabolomics: 1126 metabolites were analyzed using LC and gas chromatography MS [98], and a profile was identified that was able to distinguish between benign, clinically localized PCa and metastatic cancer. Sarcosine and the *N*-methyl derivative of the amino acid glycine were found at highly increased levels in PCa and were associated with disease progression to metastasis. However, validation of this metabolite has failed to reproduce these findings [160], and therefore, the utility of sarcosine is still under discussion.

### 2.6. Urine Biomarker Panels

Although a great number of urine biomarkers have been documented in large screening programs, there are only a few studies that take into account the heterogeneity of cancer development based on a diagnostic profile. Since a single marker may not necessarily reflect the multifactorial nature of PCa, a combination of various biomarkers in conjunction with clinical and demographic data could improve performance over the use of a single biomarker [161–163]. Adding extra genes into the “fingerprint” results in an additional layer of statistical complexity prompting new developments in biostatistics and bioinformatics [109].

Table 2 summarizes the most significant studies that have used panels of urinary biomarkers. Hessels *et al.* performed a study on 108 patients using urine sediments, where the authors combined *PCA3* with *TMPRSS2-ERG* fusion status. Combining both markers remarkably increased the sensitivity for the detection of PCa [104]. In this sense, the combination of *TMPRSS2-ERG* and *PCA3* and serum *PSA* was described as a method that could predict PCa with 80% sensitivity and 90% specificity [161] and help urologists in the decision to take PBs [162]. Furthermore, *TMPRSS2-ERG* in combination with *PCA3* enhances serum *PSA* as a marker for defining PCa risk and clinically relevant cancer on PB [163]. More recently, Lin and collaborators also combined these markers and demonstrated that they can be used to stratify the risk of having aggressive PCa [54]. Another important study came from Lexman *et al.*, who developed a multiplex model that measured the expression of seven putative PCa biomarkers and found that a combination of Golgi Membrane Protein (*GOLPH2*), Serine Peptidase Inhibitor Kazal type 1 (*SPINK1*) and *PCA3* transcript expression with *TMPRSS2-ERG* fusion status was a better predictor of PCa than *PSA* or *PCA3* alone (65.9% sensitivity and 76.0% specificity) [54]. Ouyang *et al.*, have developed a duplex qPCR assay for the detection of PCa, based on the quantification of alpha-methylacyl-CoA racemase (*AMACR*) and *PCA3* in urine sediments, while Talesa *et al.* analyzed *PSMA*, Hepsin (*HPN*), *PCA3*, UDP-n-acetyl-alpha-d-galactosamine: polypeptide *N*-acetylgalactosaminyltransferase 3 (*GalNAC-T3*) and *PSA* using qPCR and concluded that the best combination of biomarkers for predictors of PCa included urinary *PSA* and *PSMA* [117].

Rigau *et al.* [119] have developed a multiplex test based on the combination of qPCR analysis of *PCA3*, *PSGR*, *PSMA* levels in urine with serum *PSA* protein levels in a prospective study using post DRE urine samples from 57 PCa patients and 97 age-matched benign controls. They observed that by using this model, it is possible to reduce the number of unnecessary PB by 34% [119]. A multiplexed quantitative methylation-specific PCR assay consisting of three different methylated genes: *GSTP1*, *RARB* and *APC* was recently tested in a prospective multicenter study using post-DRE urine samples from 178 PCa patients and 159 controls. The predictive accuracy AUC of the assay for detecting PCa was 0.72. This was only a marginal gain in predictive ability with respect to biopsy outcome as compared to total *PSA* and DRE alone [164]. Although these combined biomarkers significantly improve sensitivity and specificity over single biomarkers, to our knowledge none of these panels have yet been established in clinical practice.

### 2.7. Exosomes as a Source of Urine Biomarkers

Exosomes are small, secreted membranous vesicles formed in multivesicular bodies through an inward budding mechanism that encapsulates cytoplasmic components [174]. For many years exosomes were thought to be organelles for the removal of cell debris or obsolete surface molecules from the cell. However, further investigations have revealed a role for exosomes in inter-cellular communication. In the last five years, several studies have demonstrated that exosomes may be secreted by multiple cell lines and cell types, including tumor cell lines, stem cells and neuronal cells [175]. In addition, exosomes have been identified in most body fluids, such as blood, urine and ascites [175]. The discovery of their nucleic acid contents, such as mRNA, small ncRNA, miRNA and mitochondrial DNA (mtDNA), which can be transported to other cells [176], represents a major breakthrough, and several studies have indicated that they can play a novel role as regulators in cell-cell communication during diverse biological processes. Urinary exosomes have recently been described as treasure chests of information and a potential source of new cancer biomarkers including PCa [15]. Analyzing the content of exosomes harvested from urine has a number of advantages: (i) it is non-invasive; (ii) data is informative with regards to PCa diagnosis and potentially the status of overall tumor malignancy; (iii) the genetic and proteomic material within exosomes is protected from enzymic degradation by the exosomal lipid bilayer [177], and (iv) exosomes are stable after long-term storage at −80 °C, which makes prospective studies feasible. Further progress has been made in terms of storage, processing [178] and analysis of protein [22] and RNA content.

To our knowledge no high-throughput technique has been used to analyze the RNA or protein content of urinary exosomes for PCa biomarker discovery in individual samples. However, some reports have indicated urinary exosomes to be an excellent source of PCa biomarkers. At a protein level, Mitchell PJ *et al*. [118] analyzed urinary exosomes from 10 healthy donors and 10 PCa patients who were undergoing hormonal therapy prior to radical radiotherapy. *PSA* and PSMA were found to be present in almost all of the PCa specimens, but not in the healthy donor specimens. At an RNA level, Nilsson *et al.* [179] showed that known RNA markers for PCa, such as *TMPRSS2-ERG* fusion transcripts and *PCA3*, could be detected in urine-derived and PCa cell line-derived exosomes by using Nested PCR [24]. This demonstrated a potential for diagnosis, as well as a strategy for the successful monitoring of the status of cancer patients. miRNAs have also been detected in extracellular fractions, stabilised by their encapsulation in microvesicles such as exosomes. Exosomes are thus a prime non-invasive source of biomarkers for cancer and other diseases [180].

## 3. Conclusions

The introduction of *PSA* testing has radically altered how PCa is diagnosed and managed. However, controversy still exists regarding both the utility of *PSA* screening for reducing PCa mortality and the risks associated with PCa over-diagnosis. Furthermore, there is the problem of the heterogeneous nature of PCa foci and problem of adequately sampling and assessing foci of poor prognosis tumor. Additional markers are therefor urgently required to supplement or replace the *PSA* test and improve the specificity of PCa detection and prognosis. Multiplex urine-based assays could provide the answer and have the advantage of potentially sampling PCa material from multiple tumor foci within individual prostates and providing both diagnostic and prognostic biomarkers [181].

It has been demonstrated that post-DRE urine samples are a rich source of biomarkers for PCa. Urine can be obtained in any urology clinic and does not require any change in routine clinical practices. Thus, post-DRE urine could be the best compromise between a minimally invasive technique and obtaining sufficient material for a correct diagnosis. However, to properly assess and validate promising urine candidates there needs to be large prospective studies of urine biomarkers using robust and standardized methods for urine collection, storage, harvest and analysis of DNA, RNA, miRNA, protein and metabolites.

**Figure 2:**
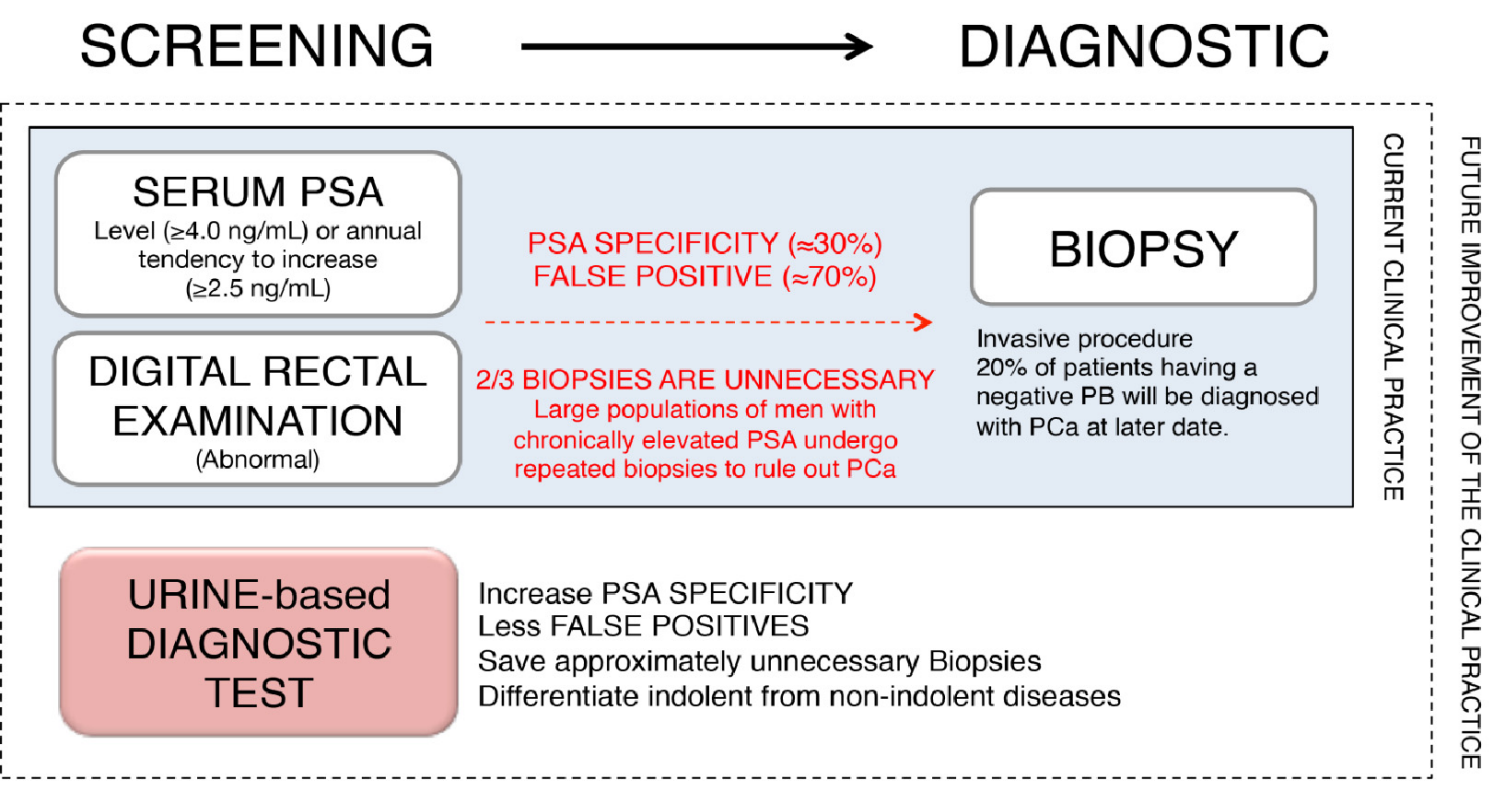
Current and future improvement in the PCa diagnostic scheme.

A future goal is therefore the development of a low cost, point of care, multiplexed, urine-based detection test for PCa which could be incorporated seamlessly into routine clinical practice to better determine which patients should undergo biopsy, and to highlight those patients that have a high risk of PCa metastasis/CRPC, and which therefor require treatment, at the earliest possible point in time (Figure 2).

In summary, the future of urine-based PCa biomarkers looks promising. It remains for us to validate the many exciting candidate biomarkers that have been discovered and to discover novel markers that will help to: (i) identify those men with indolent PCa, *i.e.*, those who will not be affected by disease in their lifetimes and who do not need treatment; (ii) minimize the number of unnecessary PBs; (iii) identify men with aggressive disease, distinguishing between who will benefit from local therapy and those who are likely to fail local therapy and require adjuvant intervention; and (iv) find markers that may serve as surrogate end points for clinical progression or survival [182].

Another important point that needs to be addressed is the necessity of the DRE. In the future, we would like to know if urine samples provided without a DRE contain enough material to correctly detect prostate biomarkers and, thus, enable a correct diagnosis. Although DRE is part of the diagnostic tripod (*PSA*, DRE and biopsy), it is usually poorly tolerated by patients and always requires medical intervention. This detail may represent a limiting factor, since the urologist would need to have the facilities to freeze and store urine samples before sending them to the laboratory. In large trials, the question of whether and how to perform the DRE to optimize sensitivity and specificity must be addressed for each potential marker [183].

## Figures and Tables

**Table 1:** Summary of PCa biomarkers in the literature.

Gene	Description	Gene type	Expression	Type of biomarker	Sample	References
*AMACR* (*P504*)	Alpha-Methylacyl-CoA Racemase	Enzyme involved in branched chain fatty acid oxidation	Over-expressed in PCa (also in HGPIN) and in some other carcinomas, both at RNA and protein level	Diagnostic (in gray zone) and prognostic	Tissue, blood and urine	[20,21]
*ANXA3*	Annexin A3	Calcium and phospholipid binding protein	Presence in urinary exosomes and proteasomes. Lower production in PCa than in BPH, HGPIN and benign	Prognostic (able to stratify a large group of intermediate-risk patients into high- and low-risk subgroups)	Tissue and urine	[22–24]
*APC*	Adenomatous polyposis coli	Tumor suppressor. Promotes rapid degradation of CTNNB1 and participates in Wnt signaling as a negative regulator.	APC methylation higher in PCa than in BPH. Methylation level correlates positively with Gleason score	Diagnostic and prognostic	Tissue and Urine DNA	[25]
*AR*	Androgen receptor	Receptor for androgen stimulation of prostate.	Over-expression associated with poor prognosis prostate cancer and metastasis	Prognostic	Tissue RNA and IHC	[26–28]
*AURKA*	Aurora kinase.	Aurora kinase. AURKA is a centrosome-associated serine/threonine kinase involved in mitotic chromosomal segregation.	Amplified and over-expressed in certain types of poor prognosis prostate cancer	Prognostic	Tissue RNA and DNA	[29–31]
*AZGP1*	Alpha-2-glycoprotein 1, zinc binding. Alias. ZAG	Stimulates lipid degradation in adipocytes and causes the extensive fat losses associated with some advanced cancers. May bind polyunsaturated fatty acids.	Over-expressed in PCa. Low AZGP1 expression predicts for recurrence in margin-positive, localized PCa	Diagnostic, prognostic	Tissue, blood and urine	[32–34]
*BRAF*	v-raf murine sarcoma viral oncogene homolog B1	Belongs to the raf/mil family of serine/threonine protein kinases and is involved in the regulation of the MAP kinase/ERKs signaling pathway, which affects cell division, differentiation.	SLC45A3-BRAF fusion gene, mutations and gain in prostate cancer	Diagnostic and therapeutic target	Tissue RNA and DNA	[35–37]
*CAMKK2*	Calcium/calmodulindependent protein kinase kinase 2.	AR target gene promoting biosynthesis and glycolysis	Down-regulation of calcium/calmodulin-dependent protein kinase kinase 2 by androgen deprivation induces castration-resistant prostate cancer.	Prognostic	Tissue RNA	[38–40]
*CDH1*	Cadherin 1, type 1, E-cadherin (epithelial)	Epithelial cell - cell adhesion molecule	Reduced production in 50% of tumors. E-cadherin production by epithelial cells has been shown to predict PCa prognosis	Prognostic (correlated with grade, tumor stage, and survival)	Tissue	[41,42]
*CLU*	Clusterin	Function unknown, but is thought to be involved in several basic biological events such as cell death and tumor progression.	Developed as a potential therapeutic target	Therapeutic target	Tissue, exosome protein	[43–46]
*CRISP-3*	Cysteine-Rich Secretory Protein 3	Secreted protein produced in the male reproductive tract, is involved in sperm maturation	Large amounts have been detected in seminal plasma. Over-expressed in HGPIN and PCa.	Prognostic	Tissue	[47,48]
*EPCA*	Early Prostate Cancer Antigen	Nuclear matrix protein	Over-expressed in PCa	Diagnostic (for predicting repeated BP)	Tissue and blood	[49,50]
*EPCA-2*	Early Prostate Cancer Antigen 2	Nuclear matrix protein	Over-expressed in PCa	Diagnostic and Prognostic (differentiate localized PCa from metastatic PCa)	Blood	[51]
*FOLH1/PS MA*	Folate hydrolase 1/Prostate Specific Membrane Antigen	Type II membrane protein. 1/N-acetylated-alpha-linked acidic dipeptidase	Over-expressed in PCa compared to BPH and normal	Diagnostic. Imaging marker and target for therapy	Tissue, blood and urine	[52,53]
*GOLM1*	Golgi membrane protein 1 (GOLPH2)	Cis-Golgi membrane protein of unknown function	Over-expressed in PCa	Diagnostic	Urine	[54,55]
*GSTP1*	Glutathione S-transferase P1	Enzyme involved in protecting DNA from free radicals	Loss of GSTP1 expression due to the promoter hypermethylation (>90% of PCa). Correlates with the number of cores found to contain PCa	Diagnostic (indicator for repeat biopsy)	Tissue and urine DNA	[56,57]
*HPN*	Hepsin	Membrane serine protease	Over-expressed in 90% PCa tumors (highly produced in HGPIN and PCa compared with BPH)	Diagnostic	Tissue	[58,59]
*IL-6*	Interleukin-6	Cytokine secreted by a variety of cell types, is involved in the immune and acute-phase response	Increased concentrations of IL-6 and IL-6R in metastatic and androgen-independent PCa	Diagnosis and Prognostic	Blood	[60–62]
*IMPDH2*	IMP (inosine 5′-monophosphate) dehydrogenase 2	Myc target gene associated with nucleotide biosynthesis	Increased serum level associated with the clinicopathological features of the patients with PCa	Diagnostic	Blood	[63]
*KLK2*	Human Kallikrein 2	Secreted serine protease	Over-expressed during PCa progression	Diagnostic and Prognostic	Tissue and blood	[64,65]
*KLK3* (*PSA*)	Kallikrein-related peptidase 3 (Prostate-Specific Antigen)	Secreted serine protease. Serum level of this protein, called *PSA* in the clinical setting, is useful in the diagnosis and monitoring of PCa.	Increased expression associated with malignant PCa	Diagnostic	Blood, urine	[66]
KLK4	Kallikrein-related peptidase 4	One of fifteen kallikrein subfamily members located in a cluster on chromosome 19	Increased expression associated with malignant PCa	Prognostic	Tissue RNA and IHC	[67,68].
MAP3K5	Mitogen-activated protein kinase kinase kinase 5	Signaling cascade	Increased expression associated with PCa	Prognostic	Tissue RNA and IHC	[69]
MKI67	Encoding antigen identified by monoclonal antibody Ki-67	Tumor growth marker, encodes a nuclear protein that is associated with and may be necessary for cellular proliferation	Increased expression associated with malignant prostate cancer	Prognostic	Tissue	[70–72]
MMP26	Matrix metallo peptidase 26	Involved in the breakdown of extracellular matrix in normal physiological processes and cancer metastasis.	Highest expression in HGPIN and decline in cancer, possible involvement in formation of early cancer.	Progression	Tissue RNA	[73–76]
MMP9	Matrix metallo proteinase 9	Implicated in invasion and metastasis of human malignancies	Over-expressed in PCa	Diagnostic	Urine	[77,78]
OR51E2/PS GR	Prostate Specific G-coupled Receptor	Receptors coupled to heterotrimeric GTP-binding proteins	Over-expressed in PCa	Diagnostic	Tissue and urine	[79–81]
PAP	Human Prostatic acid phosphatase	Enzyme	Over-expressed in PCa and in bone metastasis	Diagnostic and Prognostic of PCa bone metastasis	Blood and urine	[82,83]
PCA3	Prostate Cancer Gene 3	Non coding mRNA	Prostate specific and highly up-regulated in PCa	Diagnostic (indicator for repeat biopsy)	Tissue and urine	[84–93]
PDIA3	Protein disulfide isomerase family A, member 3.	Endoplasmic reticulum that interacts with lectin chaperones calreticulin and calnexin to modulate folding of newly synthesized glycoproteins.	Increased expression associated with malignant PCa	Prognostic	Tissue RNA and IHC	[69]
PSCA	Prostate Stem Cell Antigen	Membrane glycoprotein	Specific production in the prostate and possible target for therapy	Prognostic (correlated with higher Gleason score, higher stage, and the presence of metastasis)	Tissue and blood	[94,95]
RARB	Retinoic acid receptor, beta	Binds retinoic acid. Mediates signalling in embryonic morphogenesis, cell growth and differentiation.	DNA methylation	Prognostic	Tissue and urine DNA	[96,97]
RASSF1A	Ras association (RalGDS/AF-6) domain family member 1	Potential tumor suppressor. Required for death receptor-dependent apoptosis	DNA methylation	Prognostic	Tissue and urine DNA	[97]
Sarcosine	Sarcosine	*N*-methyl derivative of the amino-acid glycine	Seems to be differentially expressed metabolite elevated during PCa progression to metastasis	Prognostic	Urine and blood	[98]
*SPINK1*	Serine peptidase inhibitor, Kazal type 1	Serine peptidase inhibitor	Overexressed in a portion of non-ETS translocated tumors	Diagnostic	Urine, tissue	[54,99]
*TERT*	Telomerase reverse transcriptase	Maintains the telomeric ends of chromosomes and if telomerase is active, cancer cells may escape cell cycle arrest and replicative senscence	Amplification in PCa, significative association with Gleason score	Prognostic	Urine and blood	[57,100,101]
*TGFB1*	Transforming growth factor-b1	Growth factor involved in cellular differentiation, immune response, angiogenesis, and proliferation	Role of TGFβ1 in PCa progression.	Prognostic (Correlation with tumor grade and stage and lymph node metastasis)	Tissue and blood	[62,102,103]
*TIMP4*	TIMP metallopeptidase inhibitor 4	Inhibitors of the matrix metallo proteinases	Highest expression in HGPIN and decline in cancer, possible involvement in formation of early cancer.	Progression	Tissue RNA	[73–75]
*TMPRSS2:E RG*	5′ UTR of the prostate-specific androgen regulated transmembrane protease serine2 and v-ETS erythroblostosis virus E26 oncogene homolog	Gene fusion; androgen drives the expression of ETS-TF and causes tumor proliferation	The most common gene fusion in PCa. Over-expressed PCa and related to PCa aggressiveness	Prognostic for aggressive PCa and detection of PCa	Tissue and urine	[104–106]
*PLAU* and *UPAR*	Plasminogen Activator, Urokinase and Receptor	Degradation of extra cellular matrix	Over-expressed in BPH and PCa vs benign	Prognostic (increased uPA and uPAR in PCa patients with bone metastasis)	Tissue and blood	[107,108]

**Table 2:** Summary of the most significant studies that have used panels of urine biomarkers for PCa detection.

Biomarker type	Study	Marker	PCa/study	Sens.	Spec.	AUC
DNA	Hoque *et al.*, 2005 [112]	*p16, ARF, MGMT, GSTP1*	73	87%	100%	ND
	Rouprêt *et al.*, 2007 [110]	*GSTP1, RASSF1A, RARB,* and *APC*	95/133	87%	89%	ND
	Vener *et al.*, 2008 [165]	*GSTP1, RARB* and *APC*	54/121	55%	80%	0.69
	Payne *et al.*, 2009 [166]	*GSTP1, RASSF2, HIS1H4K, TFAP2E*	192	94%	27%	
	Baden *et al.*, 2009 [164]	*GSTP1, RARB* and *APC*	178/159	ND	ND	0.72
	Costa *et al.*, 2011 [113]	*PCDH17, TCF21*	318	26%	100%	

mRNA	Hessels *et al.*, 2007 [104]	*PCA3* and *TMPRSS2:ERG*	78/108	73%	52%	ND

	Laxman *et al.*, 2008 [54]	*PCA3, GOLPH2, SPINK1* and *TMPRSS2:ERG*	152/257	66%	76%	0.76

	Ouyang *et al.*, 2009 [167]	*AMACR* and *PCA3*	43/92	72%	53%	ND

	Talesa *et al.*, 2009 [117]	*PSMA, HPN, PCA3, GalNAC-T3* and serum *PSA*		49%	ND	ND

	Rigau *et al.*, 2010 [81]	*PCA3 and PSGR*	73/215	96%	34%	0.73

	Rigau *et al.*, 2011 [119]	*PSMA, PSGR, PCA3* and serum *PSA*	57/154	96%	50%	0.82

	Salami *et al.*, 2011 [168]	*PCA3, TMPRSS2:ERG* and serum *PSA*	15/45	80%	90%	0.88

	Jamasphvili *et al.*, 2011 [169]	*PCA3, AMACR, TRMP8, SMSB*	104	72%	71%	
	Nguyen *et al.*, 2011 [170]	*TMPRSS2:ERG* subtypes	101	35%	100%	

	Tomlins *et al.*, 2011 [171]	*PCA3* and *TMPRSS2:ERG*	463 (acad.) and 439 (biopsy)	a_0.64 and b_0.66		

Protein	Rehman *et al.*, 2004 [154]	*ENO1, IDH3B, B2M, A1M, PRO2044* and *S100A9* (Calgranulin*_B/MRP-14*)	6 PC (12)			

	Theodorescu *et al.*, 2005 [155]	Proteinpolypeptide	26/47	92%	96%	

	M’Koma *et al.*, 2007 [157]	130 m/z	89/407	81%	80%	

	Theodorescu *et al.*, 2008 [156]	12 protein pannel + age + serum *PSA*	86 Training set + 213 validation set	91%	62%	

	Okamoto *et al.*, 2008 [158]	72 masspicks	57/113	91%	83%	

Mixture	Cao *et al.*, 2010 [172]	mRNA, protein and metabolite (*PCA3, TMPRSS2: ERG*, *ANXA3*, Sarcosine, and urine *PSA*)	86/131	95%	50%	0.86

	Prior *et al.*, 2010 [173]	mRNA (*AMACR/MMP2),* DNA (*GSTP1/RASSF1A*) and *PSA* in serum and urine	34/113	57%	97%	0.79
